# Neurological Applications of Celery (*Apium graveolens*): A Scoping Review

**DOI:** 10.3390/molecules28155824

**Published:** 2023-08-02

**Authors:** Terence Yew Chin Tan, Xin Yi Lim, Nor Azrina Norahmad, Hemahwathy Chanthira Kumar, Bee Ping Teh, Nai Ming Lai, Ami Fazlin Syed Mohamed

**Affiliations:** 1 Herbal Medicine Research Centre, Institute for Medical Research, Ministry of Health, Shah Alam 40170, Malaysia; 2 School of Pharmacy, Monash University Malaysia, Subang Jaya 47500, Malaysia; lainm123@gmail.com; 3 School of Medicine, Taylor’s University, Subang Jaya 47100, Malaysia

**Keywords:** celery, *Apium graveolens*, 3-n-butylphthalide, NBP, central nervous system, neurological disorders, herbal medicin

## Abstract

*Apium graveolens* is an indigenous plant in the family Apiaceae, or Umbelliferae, that contains many active compounds. It has been used traditionally to treat arthritic conditions, gout, and urinary infections. The authors conducted a scoping review to assess the quality of available evidence on the overall effects of celery when treating neurological disorders. A systematic search was performed using predetermined keywords in selected electronic databases. The 26 articles included upon screening consisted of 19 in vivo studies, 1 published clinical trial, 4 in vitro studies and 2 studies comprising both in vivo and in vitro methods. *A. graveolens* and its bioactive phytoconstituent, 3-n-butylphthalide (NBP), have demonstrated their effect on neurological disorders such as Alzheimer’s disease, Parkinson’s disease, stroke-related neurological complications, depression, diabetes-related neurological complications, and epilepsy. The safety findings were minimal, showing that NBP is safe for up to 18 weeks at 15 mg/kg in animal studies, while there were adverse effects (7%) reported when consuming NBP for 24 weeks at 600 mg daily in human trials. In conclusion, the safety of *A. graveolens* extract and NBP can be further investigated clinically on different neurological disorders based on their potential role in different targeted pathways.

## 1. Introduction

Neurodegenerative illnesses are defined as a loss of functionality and the eventual death of nerve cells in the brain or peripheral nervous system [1]. One in three people are estimated to experience a neurological condition at some point in their lives, making them the second largest cause of mortality and the primary source of disability [2,3]. Most available prevalence data are focused on dementia, as it is the highest contributing factor towards neurodegenerative diseases. However, apart from the most common neurodegenerative diseases such as Parkinson’s disease, Alzheimer’s disease, multiple sclerosis, and stroke [4], there are also a wide range of other neurological diseases such as prion disease, motor neuron diseases, Huntington’s disease, spinocerebellar ataxia and spinal muscular atrophy [5]. Anatomical (functional systems), cellular (neuronal groups), protein vulnerability (structural change, biochemical modifications and altered physiological function), and genetic changes all affect how these diseases develop. Persistent neuroinflammation often occurs, and a neurological disease’s pathogenesis is often complex, with all these factors interlinking and perpetuating each other [6]. Current therapeutic options for neurological diseases mostly provide symptomatic support for the patients and caregivers, while a successful cure is yet to be found. Early diagnosis is essential for treatment planning and can help to optimize support for patients and their families in the long run [7]. Recent reviews have been published regarding the use of herbal medicine for the treatment of neurodegenerative diseases [8,9]. Therefore, herbal treatments should be considered a potential therapeutic candidate in order to tackle neurological disorders.

Celery is among the plants that have recently gained popularity in research [10,11]. Celery (*A. graveolens*) is an indigenous plant of the family Apiaceae, or Umbelliferae, originating in the Mediterranean [12,13]. It is most easily identified by its thick, very erect stem. It is used as a food in most parts of the world. Celery contains many active compounds, including polysaccharides (apiuman) [14], flavonoids (luteolin, apigenin) [15], phthalides (sedanolide, 3-n-butyl phthalide) [16,17], furanocoumarins (bergapten, xanthotoxin) [18], terpenes (d-limonene) [17], amino acids (L-tryptophan) [16], polyacetylenes (falcarinol, falcarindiol) [19,20], and vitamins (alpha-tocopherol) [21]. One of its bioactive compounds, known as butylphthalide, which, is a light-yellow viscous compound comprising a family of optical isomers that includes l-3-*N*-butylphthalide (L-NBP), d-3-*N*-butylphthalide (D-NBP), and dl-3-*N*-butylphthalide (DL-NBP), is known for its therapeutic value. Based on its traditional use, *A. graveolens* has been known to relieve joint pain, gout, and urinary infections [22]. It has also been used traditionally to increase urine excretion, promote menstrual discharge, and treat dengue fever and inflammation or pain in muscles or joints [23]. Based on in vivo or in vitro studies, *A. graveolens* has shown its pharmacological efficacy with antimicrobial, antifungal, anti-parasitic, anti-inflammatory, anti-cancer, anti-ulcer, antioxidant, anti-diabetic, anti-infertility, anti-platelet, anti-spasmolytic, hepatoprotective, cardioprotective, neuroprotective, cytoprotective, hypolipidemic, and analgesic activity [24]. There is a need to review all relevant studies to assess whether celery has an effect on neurological disorders. Despite the growing evidence, there have been no known published systematic or scoping reviews narrating the effect of celery when treating neurological disorders. Therefore, this scoping review aimed to collate and assess the quality of the currently available scientific evidence on the overall potential use of celery in neurological disorders.

## 2. Results

### 2.1. Study Inclusion

**Figure 1:**
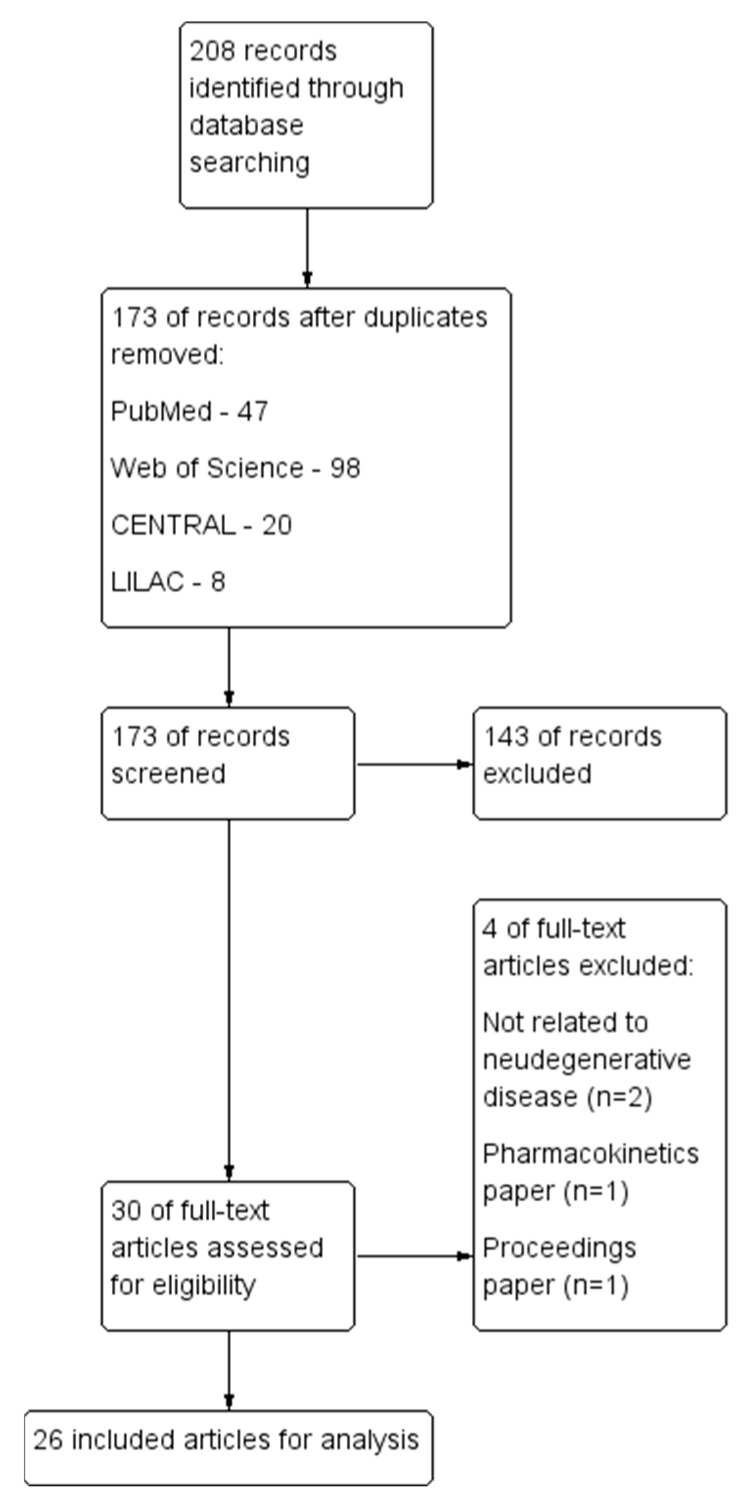
Preferred Reporting Items for Systematic Reviews and Meta-Analyses (PRISMA) flowchart.

A total of 208 records were identified from the initial search, with a final 26 articles included [25,26,27,28,29,30,31,32,33,34,35,36,37,38,39,40,41,42,43,44,45,46,47,48,49,50]. One clinical study was identified, while the rest were 19 in vivo studies, 4 in vitro studies, and 2 studies employing both in vivo and in vitro methods. The study selection process is presented in the Preferred Reporting Items for Systematic Reviews and Meta-Analyses (PRISMA) flowchart (Figure 1).

### 2.2. Characteristics of Included Studies

Overall, the included studies focused on the efficacy of *A. graveolens*, with the majority of studies investigating NBP as the main phytoconstituent and its derivatives and analogues (n = 20); this was followed by extracts (n = 5), with one study not describing the intervention in sufficient detail. The extracts were mostly sourced from the whole plant or the aerial parts of *A. graveolens*, while a majority did not mention the source of the NBP and its derivatives. Among the included studies, the in vivo studies were mainly focused on Alzheimer’s disease (n = 4) and stroke-related neurological complications (n = 4), followed by depression (n = 3), the general mechanisms of action of neurological disorders (n = 3), diabetes-related neurological complications (n = 2), epilepsy (n = 2) and others (Parkinson’s disease (n = 1), anxiolytics (n = 1), and neurotoxicity (n = 1)). In vitro studies were mostly on Parkinson’s disease (n = 3), followed by others (diabetes (cognitive decline) and stroke in support of in vivo findings (n = 2), and Charcot–Marie–Tooth disease (n = 1)). The clinical study focused on therapy for Parkinson’s disease (n = 1).

Among the included studies, 4 out of 26 underwent the authentication process through the voucher specimen deposition of the plant. In total, 4 out of 26 studies reported the use of a qualitative analysis to determine the phytochemicals associated with *A. graveolens*. In total, 3 out of 26 studies performed a quantitative analysis in order to determine the composition of the associated phytochemicals in *A. graveolens.* Only one study reported [28] using a standardized formulation of the methanolic extract of the whole *A. graveolens* plant. The routes of administration of the intervention included oral, intranasal, intravenous, and intraperitoneal. Detailed information on the qualitative and quantitative phytochemical analysis, as well as the standardization formula of the herbal interventions of all included studies, are presented in the Supplementary Material: Table S3.

### 2.3. In Vivo Studies

All of the 19 studies were in vivo studies, and 2 were supported by additional in vitro findings, which further explored potential mechanisms of action. Four studies conducted between the years 2010 and 2016 focused on Alzheimer’s disease using L-3-n-butylphthalide (L-NBP), with an oral dosage of 15 mg/kg for a treatment duration of three months or more. The findings showed that L-NBP improved synaptic functions; reduced Aβ plaque load, oxidative stress, and microglia activation; and inhibited abnormal tau hyperphosphorylation [33,34,39,45].

Another four studies were focused on stroke-related neurological disorders such as cerebral ischemia reperfusion, focal ischemic stroke, and intracerebral hemorrhage using either DL-3-n-butylphthalide (DL-NBP) or L-NBP; the study employed various doses and routes of administration (intranasal, intraperitoneal and intragastric) for a duration of 2 to 14 days. The findings showed that DL-NBP significantly decreased neurological deficit scores and increased the diameter of collaterals (arteriogenic effect), while L-NBP inhibited the expression of tumor necrosis factor-alpha (TNF-α) and matrix metallopeptidase 9 (MMP-9), thereby reducing inflammatory reactions due to intracerebral hemorrhage [30,37,43,44].

For depression-related neurological disorders, three studies used a crude 70% methanolic extract of *A. graveolens* or DL-NBP with dosages between 10 mg/kg and 500 mg/kg, administered either orally or intraperitoneally for a durations of two weeks to six weeks. The findings for the methanolic extract of *A. graveolens* showed a significant improvement in immobility and the climbing times at all treatment intervals, comparable with the fluoxetine treatment. In terms of the congnitive-enhancing effects using Morris water maze and object recognition tests, *A. graveolens* increased the novel exploration time more than the donepezil treatment (*p* < 0.05) in a non-dose-dependent manner [26]. The DL-NBP showed significant findings with regard to the increased locomotor activity; the increased sucrose preference in the sucrose preference test; the decreased immobility time in the forced swimming test; and the increased number of crossing and rearing behaviors in the open-field test [27,40].

An aqueous extract of *A. graveolens* and DL-NBP were studied for diabetic-related neurological disorders between 20 mg/kg and 120 mg/kg for four to eight weeks. DL-NBP showed neuroprotective effects in diabetes-associated cognitive decline through hippocampal morphology normalization, by improving synaptic plasticity, and by reducing neuronal apoptosis [36]. However, there was no mention of the dosage and duration of the administration of the *A. graveolens* aqueous extract, which demonstrated only positive results in the step-through latency test, with no significant improvements in the initial latency and Y-maze test [35].

With regard to celery’s role in epilepsy, two studies [29,41] using either an aqueous extract of *A. graveolens* or L-NBP at doses between 80 mg/kg and 1000 mg/kg administered intraperitoneally showed increased minimal clonic seizure (MCS) latency and a significant amelioration of epileptiform activity (*p* < 0.05) compared to the saline or vehicle (tween-80) based on electroencephalography readings.

Other studies relating to celery’s potential in neurological disorders include those focusing on Parkinson’s disease, neurotoxicity, chronic cerebral hypoperfusion, chronic intermittent hypoxia hypercapnia, anxiolytics, and perinatal effects [25,28,31,32,38,42]. Most of its protective effects were related to improving oxidative stress [25,28,38] and the inhibition of apoptosis or neuronal death via the upregulation of the CNTF/CNTFRa/JAK2/STAT3 signaling pathway [31], the activation of the SIRT1/PGC-1a signaling pathway [32] or the upregulation of the TGF-β1/Akt/Wnt/β-catenin pathway [42]. The scientific evidence for the pharmacological properties of *A. graveolens* and its phytoconstituent are described in the tables and narratively, as follows (Table 1):

### 2.4. Risk of Bias Assessment of In Vivo Studies

**Figure 2:**
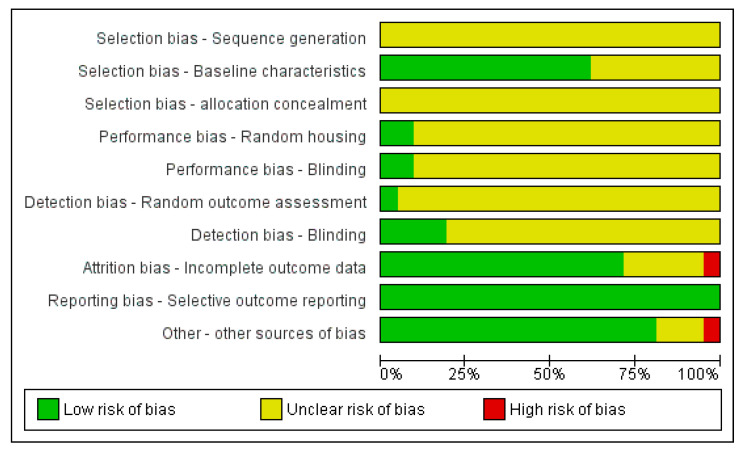
Risk of bias summary of included in vivo studies.

**Figure 3:**
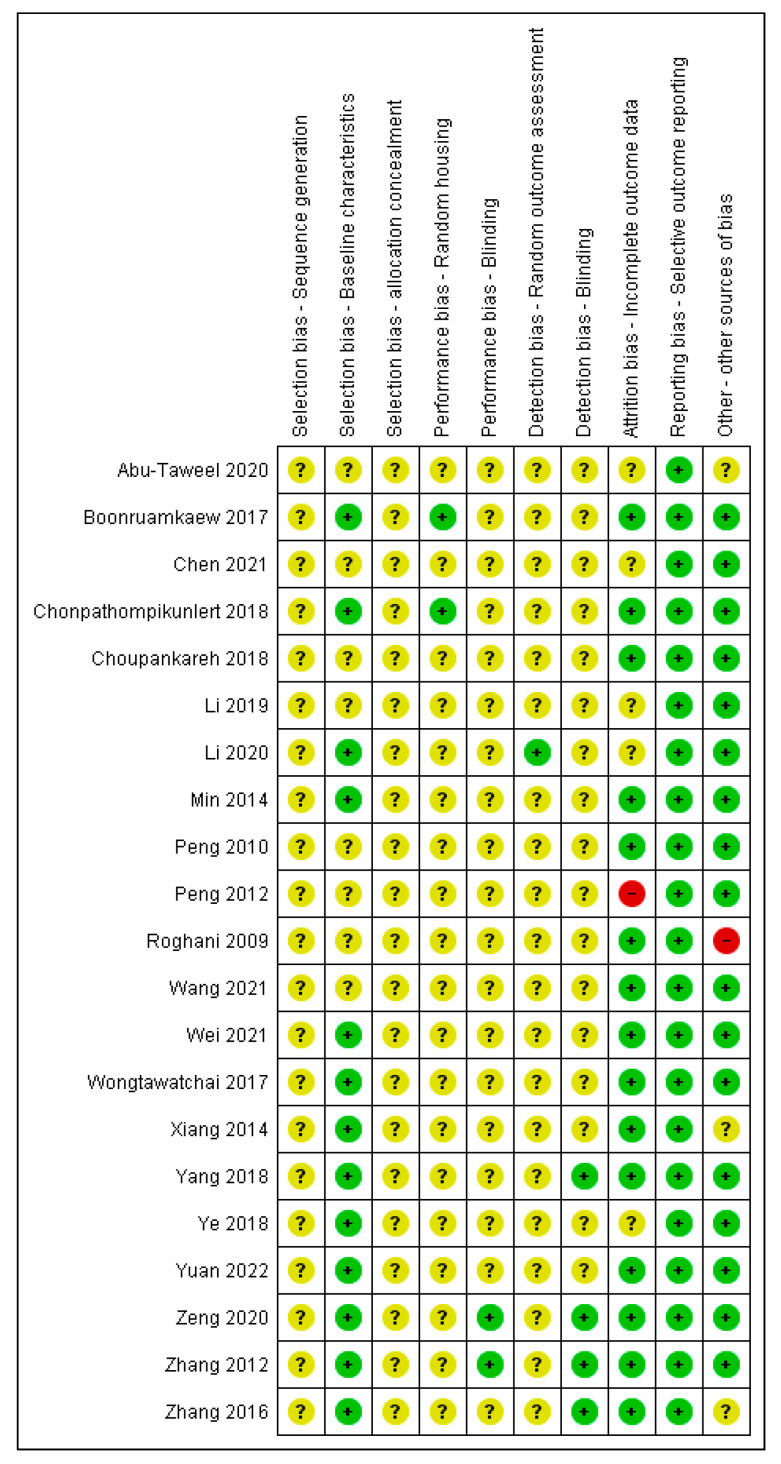
Risk of bias assessment of included studies (in vivo) of *A. graveolens* for neurological disorders. Note: Green for low risk of bias, yellow for unclear risk of bias and red for high risk of bias [25,26,27,28,29,30,31,32,33,34,35,36,37,38,39,40,41,42,43,44,45].

Figure 2 and Figure 3 show the risk of bias assessment results for the 21 included in vivo studies. The majority of studies have an unclear risk of bias in random sequence generation, allocation concealment, random housing, blinding, and the blinding of the outcome assessment, as all these studies did not report on these domains.

All studies (100%) showed a low risk of bias in selective reporting, while more than 70% of the studies showed a low risk of bias for the baseline characteristics and attrition bias (as incomplete outcome data). A further 10% of the studies were assessed as having a high risk for other biases due to a lack of details regarding the origins of the test item and the study funding.

### 2.5. In Vitro Studies

In total, 4 out of the 26 included studies were in vitro studies and 2 were additional in vitro findings from in vivo studies that further explored potential mechanisms of action. Most in vitro studies (n = 3) focused on Parkinson’s disease in relation to neurological disorders. NBP and its racemic (L-NBP/DL-NBP) showed protective effects in Parkinson’s disease cell models through reducing cytotoxicity, preserving the dendritic processes surrounding cells, decreasing apoptotic cells, and inhibiting tau protein hyperphosphorylation. [46,47,49]. Another two studies [36,37] were supportive in vitro findings to the in vivo studies for exploring the mechanisms of action in stroke and diabetes (cognitive decline) models. One study [48] analyzed the effects of L-NBP on a hereditary disease known as Charcot–Marie–Tooth disease (CMT), which harms the peripheral nerves. The scientific evidence of the pharmacological properties of *A. graveolens* and its phytoconstituents are described in the tables and narratively, as follows (Table 2):

### 2.6. Clinical Trial

One clinical trial was included. It is was prospective, single-center, parallel-group, randomized controlled trial using DL-NBP as therapy for Parkinson’s disease. Patients with idiopathic Parkinson’s disease were treated with 200 mg of DL-NBP, thrice daily for 24 weeks, alongside concomitant existing medications that patients were already taking. The findings showed improvements in symptoms such as bradykinesia plus stiffness, based on the non-tremor score, sleep quality, via the Pittsburgh sleep quality index scores, and quality of life, by NBP therapy [50].

### 2.7. Safety Study

In total, 3 out of 26 studies contained safety findings; these were in vivo studies (n = 2) [33,45] and one clinical trial [50]. One study using L-NBP at 15 mg/kg showed no significant toxicity in mice after monitoring their general health for 18 weeks [33]. However, another study using L-NBP with a similar dose for 18 weeks reported that the mice gradually died due to the poor physical condition of aging [45]. For the clinical trial [50], it was reported that 3 adverse events out of 43 were directly associated with the treatment in the NBP group at 200 mg three times a day for six months; these events included itching and skin rash (n = 1), a slight elevation in the levels of alanine transaminase (ALT) enzyme, and a mild gastrointestinal reaction.

## 3. Discussion

According to the Pan American Health Organization, neurological disorders account for 533,172 deaths, 7.5 million years of life lost due to premature mortality, and 8.2 million years lived with disability [51]. The included studies show that *A. graveolens* and its compounds have potential applications in various neurological disorders, although most of the reported studies were in the in vivo stage.

### 3.1. Parkinson’s Disease

Parkinson’s disease is the only application of the NBP compound, instead of a plant extract, that has successfully reached the clinical trial stage. NBP has been shown to improve behavioral abnormalities in a Parkinson’s disease mice group; reduce oxidative stress via reducing malondialdehyde levels and increasing glutathione peroxidase and the percentage inhibition of oxygen; and protect the dopaminergic neurons by reducing the activity of monoamine oxidase types A and B [28]. These findings were further supported by a study that combined DL-NBP with mesenchymal stem cells, showing enhanced neuroprotection in Parkinson’s disease caused by concussive head injury [52].

### 3.2. Alzheimer’s Disease

In Alzheimer’s disease studies, L-NBP has been shown to improve synaptic functions; reduce Aβ plaque load, oxidative stress, and microglia activation; and inhibit abnormal tau hyperphosphorylation, which plays a role in the Aβ tau synergy [45]. It is now thought that there are two different types of interactions: major physical interactions between the two proteins at the synapse, or indirect interactions caused by Aβ and tau’s effects on neuronal physiology (activating kinases, preventing tau degradation, regulating excitability and gene expression, and activating glia) in slowing the progression of Alzheimer’s disease [53]. Celery’s potential in treating Alzheimer’s disease needs to be further assessed by capturing the spatiotemporal progression of Aβ and tau pathology and other disease characteristics, as well as considering the contribution of complex genetic and environmental variables that influence disease phenotypes [53].

### 3.3. Stroke-Related Neurological Disorders

In stroke, NBP has potential with its dual role; it has arteriogenic effects and the ability to inhibit the expressions of TNF-α and MMP-9. Arteriogenic effects may benefit the maintenance of pial collaterals, which are small arterial connections joining the terminal cortical branches of the major cerebral arteries along the surface of the brain, and are therefore important in supporting a functional brain environment [54]. Conversely, TNF-α production is triggered by ischemia during stroke as an inflammatory response, leading to the activation of MMP-9 expression related to secondary bleeding in the BBB [55]. These collectively show the pleiotropic effects of NBP, which may be beneficial, given that the pathogenesis of stroke is multifactorial and involves multiple pathways of neuroexcitotoxicity, neuroinflammation, structural damages, oedema in the BBB, oxidative damages, as well as overall neurodegeneration.

### 3.4. Other Neurological Disorders

Other targeted pathways reported for the effects of *A. graveolens* in neurological disorders include Nrf2 and NF-κB pathways; BDNF/ERK/mTOR (antidepressant); CNTF/CNTFRa/JAK2/STAT3 (cerebral blood flow decreases); and SIRT1/PGC-1a (obstructive sleep apnea). All these signaling pathways are essential contributors to chronic neuroinflammation and oxidative stress in the brain. To further understand the complex transcriptional regulation of brain function in various disease models, in-depth research on the effects of celery and its bioactive constituents and their temporal effects on upstream regulators and downstream effector signaling pathways in neuroinflammation and neuronal damage needs to be carried out [56].

### 3.5. Celery’s Mechanisms of Action in Neurological Diseases

**Figure 4:**
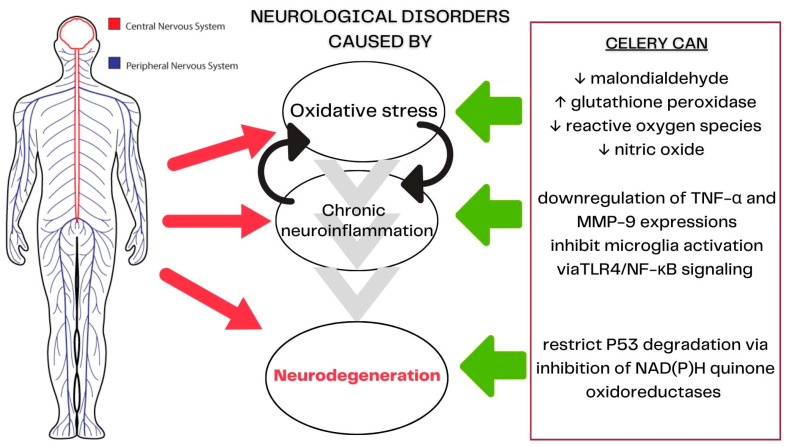
Role of celery in treating neurological disorders.

Celery and its bioactive compound play a role in oxidative stress, inflammatory responses, and neuronal apoptosis [57]. Most neurological disorders have three common underlying mechanisms. The first is oxidative stress, which causes cellular damage involving Nrf2 [58]. NBP is a known potent antioxidant in activating the Nrf2 enhanced expressions of antioxidant enzymes [59,60]. These enzymes will reduce reactive oxygen species (ROS) and prevent mitochondrial damage [61,62]. The second central mechanism is prolonged and unregulated neuroinflammation related to NF-κB, with the production of pro-inflammatory cytokines and chemokines associated with the self-potentiation of the neuroinflammatory cycle. NBP plays a role in the downregulation of TNF-α and MMP-9 expressions, leading to the inhibition of microglia activation via TLR4/NF-κB signaling, and reducing inflammation [63,64,65]. The third pathway involves neurodegeneration through apoptosis, autophagy, and necrosis. Neurodegeneration is significantly influenced by oxidative stress and chronic neuroinflammation through the regulation of p53 activity. In a molecular docking study, NBP showed its potential to suppress glial apoptosis by limiting p53 degradation by inhibiting NAD(P)H quinone oxidoreductases [66]. In addition, an animal study of Alzheimer’s disease supported NBP’s role in decreasing the expression of p53 in the cortex, improving learning and memory abilities [67]. The role of celery’s action in neurological diseases is summarized in Figure 4.

### 3.6. Limitations

Most of the included studies have a similar limitation. Although the findings of these studies support the role of *A. graveolens* in signaling pathways, they lack in understanding the molecular mechanism that contributes towards celery’s neuroprotective and pharmacological effect. For example, NBP was studied on its neuroprotective role in BBB disruption following ischemic stroke without conducting an in-depth study of the internal relationship, possible targets, or effect of the molecular mechanism of NBP on protecting the BBB after cerebral ischemic reperfusion [30]. When considering potential therapeutic candidates for diseases of the central nervous system, the route of administration used to deliver the drug to the brain is an important consideration. This is to ensure that the drug interaction occurs on the specific targeted site. Based on our included studies, the routes of administration include oral, intranasal, intravenous, and intraperitoneal. A distribution study that evaluated the metabolic profile of NBP in rats via a radiochromatograph showed that the delivery of NBP from the blood to the brain is limited by the BBB [68]. Therefore, much research is needed to develop herb-based formulations with improved delivery using exosomes, nanoparticles, active transporters or brain permeability enhancers, and other non-invasive techniques [69]. As this is a scoping review, we did not perform meta-analyses of the data. However, with time, when sufficiently homogenous literature is present for any single neurological disorder, systematic reviews with meta-analyses may be performed in the future.

## 4. Materials and Methods

A scoping review of the literature was conducted in accordance with the methodology by Levac et al. [70]. The Preferred Reporting Items for Systematic reviews and Meta-Analyses extension for Scoping Reviews (PRISMA-ScR) guidelines were followed, which are a set of 20 essential items and 2 optional items that were created to help improve the quality, completeness, and transparency of scoping reviews; this is presented in the Supplementary material: Table S1 [71].

### 4.1. Review Objective

This scoping review was conducted to evaluate the worldwide scientific evidence on the pharmacological properties and safety of *A. graveolens* plant for neurological disorders.

### 4.2. Inclusion and Exclusion Criteria

#### 4.2.1. Type of Study

This review considered both clinical and preclinical (in vivo, in vitro) articles. Proceeding articles were excluded due to a lack of information for critical appraisal.

#### 4.2.2. Type of Participants

This review included studies that either recruited human subjects with any neurological disorder, animal models or cell studies, all addressing both central and peripheral systems.

#### 4.2.3. Type of Intervention

This review considered any form of *A. graveolens*, including all plant parts and preparations including crude preparations, extracts, standardized extracts, finished products in pharmaceutical forms (e.g., capsule, tablets, powder, liquid) containing *A. graveolens* as a sole active ingredient, as well as its representative compounds.

To assess celery as a whole or as its constituents, as well as a single contributing intervention, this review excluded studies using co-intervention in combination with celery.

#### 4.2.4. Type of Outcomes

The following primary and secondary outcomes were selected prior to screening and the selection of studies to facilitate a systematic assessment of the outcome measures. These outcomes were selected based on the effects of the compounds on central and peripheral nervous system disorders found in a published literature review and general web search [72,73,74,75,76,77].

##### Primary Outcomes

Pharmacological properties of *A. graveolens* in neurological disorders.

Preclinical and clinical outcomes of *A. graveolens* efficacy studies.

Mechanism of action of *A. graveolens* in efficacy studies.

##### Secondary Outcomes

Safety: this included adverse events and safety monitoring information from clinical studies, as well as toxicity and safety pharmacology studies from animals that were related to applications in neurological disorders.

### 4.3. Search Strategy

The electronic databases MEDLINE, Web of Science, LILAC, and Cochrane Central Register of Controlled Trials (CENTRAL) were searched for published studies from inception until November 2022. There were no restrictions applied in terms of the publication period and language. In addition to database searches, the team screened the reference lists and citations of retrieved articles to further identify studies for inclusion. In cases of ambiguity, attempts were made to contact the authors of relevant articles that met the inclusion criteria for this review.

The search strategies (Supplementary Table S2) were translated into the other databases using the appropriate controlled vocabulary, as applicable. The general search terms used were celery and neurological disorders and their synonyms.

### 4.4. Study Selection

A pair of review authors independently screened titles and abstracts from the search strategy according to the inclusion and exclusion criteria, with disagreements resolved via discussion, with the help of a third author as an arbiter if required.

### 4.5. Data Extraction and Management

A pair of review authors independently coded all data from each included study using a pro forma designed specifically for this review. The interventions defined in the study were compared against our pre-defined intervention. Any disagreement among the review authors was resolved by discussion leading to a consensus, with referral to a third review author if necessary.

### 4.6. Data Analysis

#### Risk of Bias Assessment

Two review authors (XYL, TT) independently assessed each article included for risk of bias in animal studies using the Systematic Review Centre for Laboratory animal Experimentation (SYRCLE) risk of bias tool. These authors scored the risk of bias in each domain and the overall risk was reported using the Cochrane Review Manager (RevMan, version 5.4) software [78]. Any disagreement among the review authors was resolved by discussion leading to a consensus and involved a third review author if necessary.

## 5. Conclusions

In conclusion, *A. graveolens,* especially its phytoconstituent NBP, can be further investigated regarding different neurological disorders based on its potential to have pleiotropic effects on different targeted pathways for neurological pathogenesis. The safety of celery extracts and NBP needs to be further established with better quality standards of reporting for a meaningful evaluation of its dosage, efficacy, and safety before its application in future clinical trials.

## Figures and Tables

**Table 1:** Pharmacological properties of *Apium graveolens* in neurological disorders (in vivo).

Animal	Intervention Details	Disease Model	Dosage, Duration, Route	Effect/Mechanism	Reference
Rat	Details of celery not mentioned	Perinatal effect	300 or 600 mg/kg, 15 days, oral	Protective effects of celery against various lipopolysaccharide-induced oxidative stresses	Abu-Taweel, 2020 [25]
Mice	Crude 70% methanol extract of *A. graveolens*	Depression	65, 125, 250, 375 and 500 mg/kg, 4 weeks, oral	Antidepressant-like effects of *A. graveolens* in the forced swimming and tail suspension tests, and the cognitive-enhancing effect validated in the Morris water maze and object recognition tests	Boonruamkaew, 2017 [26]
Rat and mice	DL-NBP	Depression	Rats: 10, 30, 100 mg/kg Mice: 30 mg/kg 6 weeks, oral	Antidepressant effect of DL-NBP via activation of BDNF/ERK/ mTOR cascade in the cortex and involvement of serotonergic system	Chen, 2021 [27]
Mice	70% methanol crude extract of *A. graveolens* whole plant	Parkinson’s disease	125, 250, 375 mg/kg, 21 days, oral	Amelioration of behavioral impairments, improvement in oxidative stress parameters, decrease in the activity of MAO-A and B, and protection of dopaminergic neurons by celery extract	Chonpathompikunlert, 2018 [28]
Rat	Aqueous extract of *A. graveolens* aerial part	Epilepsy	100, 500, and 1000 mg/kg, 30 min, i.p	*A. graveolens* extract possesses anticonvulsant activity and is accompanied by an antioxidant effect in the brain	Choupankareh, 2018 [29]
Mice	DL-NBP	Ischemic stroke	40 mg/kg, duration and route not mentioned	DL-NBP NBP exerts its neuroprotective effects through attenuating the cerebral infarct size and neurological deficit score, reducing cerebral edema and BBB permeability	Li, 2019 [30]
Rats	DL-NBP	Chronic cerebral hypoperfusion (CCH)	5 mg/kg, once daily, 21 days, intravenous	DL-NBP administration markedly rescues memory deficits and hippocampal neuronal death/ apoptosis by upregulating the CNTF/CNTFRa/JAK2/STAT3 signaling pathway in CCH rats	Li, 2020 [31]
Rats	DL-NBP	Chronic intermittent hypoxia hypercapnia (CIHH)	80 mg/kg, 2 weeks, oral	Neuroprotective effects of DL-NBP under CIHH condition possibly occurring through the inhibition of apoptosis, promotion of hypoxia-induced autophagy, and activation of the SIRT1/PGC-1a signalling pathway	Min, 2014 [32]
Mice	L-NBP	Alzheimer’s disease	15 mg/kg, 5 days/week, 18 weeks, oral	L-NBP reduces cerebral Aβ levels, glial activation, oxidative stress, cognitive impairment; regulates APP processing toward the nonamyloidogenic pathway; and promotes APP release, thereby precluding Aβ generation.	Peng, 2010 [33]
Mice	L-NBP	Alzheimer’s disease	15 mg/kg, 5 days/week, 12 weeks, oral	L-NBP is able to inhibit tau abnormal hyperphosphorylation and improve cognitive impairment in an APP/PS1 transgenic	Peng, 2012 [34]
Rat	Aqueous extract of *A. graveolens*	Diabetes (learning memory)	Dosage, and route of administration not mentioned, 4 weeks	Chronic oral administration of celery could enhance the consolidation and recall capability of stored information and does not affect spatial memory	Roghani, 2009 [35]
Mice	DL-NBP	Diabetes (cognitive decline)	20, 60, 120 mg/kg, 8 weeks, oral	DL-NBP shows neuroprotective effects and inhibits cognitive impairment in diabetes by normalizing hippocampal morphology, improving synaptic plasticity, and reducing neuronal apoptosis	Wang, 2021 [36]
Mice	DL-NBP	Stroke	5 µL (total 80 mg/kg in 400 μL vegetable oil), 1 h after the stroke onset and once daily, 14 days, intranasal	DL-NBP has potential arteriogenic effects for stroke treatment through restoration of local cerebral blood flow and other sustainable positive outcomes	Wei, 2021 [37]
Rat	Methanol extract of *A. graveolens* whole plant	Anxiety	125 and 250 mg/kg, 3 weeks, oral	Methanol extract of *A. graveolens* has protective effect against immobilization (stress-induced anxiety-like behavior) without memory loss.	Wongtawatchai, 2017 [38]
Mice	L-NBP	Alzheimer’s disease	10 and 30 mg/kg, 4 weeks, route of administration not reported	L-NBP significantly increases the expression of BDNF/TrkB/PI3K/AKT, in the brain improving cognitive impairment	Xiang, 2014 [39]
Rat	DL-NBP	Depression	30 mg/kg, 14 days, oral	DL-NBP has antidepressive effects involving the Nrf2 and NF-κB pathways responsible for neuroinflammation and oxidative stress	Yang, 2018 [40]
Mice	L-NBP	Epilepsy	80 mg/kg, 14 days, intraperitoneal	L-NBP reduces seizure severity and aberrant electroencephalogram	Ye, 2018 [41]
Rat	NBP	Neurotoxicity	40 and 80 mg/kg, 22 days, oral	NBP administration could mitigate the motor and cognitive impairment caused by neurotoxicity and mitochondrial damage	Yuan, 2022 [42]
Rat	L-NBP	Stroke	50 mg/kg, duration not clear, intraperitoneal	L-NBP inhibits the expression of TNF-α and MMP-9 reducing inflammation, BBB damage and intracerebral hemorrhage	Zeng, 2020 [43]
Rat	DL-NBP	Stroke	60 mg/kg (pre-treatment); 80 mg/kg (post treatment), 2 months before stroke-induced (pre-treatment), 1 week post (post-treatment), intragastric	DL-NBP exerts both preventive and therapeutic effects on ischemic stroke in hypertensive rats, but only exerts therapeutic effects in normotensive rats	Zhang, 2012 [44]
Mice	L-NBP	Alzheimer’s disease	15 mg/kg, 12 weeks, oral	L-NBP enhances synaptic performance, decreases Aβ plaque load, and inhibits microglia activation	Zhang, 2016 [45]

Abbreviation. BBB: Blood–brain barrier; BDNF: Brain-Derived Neurotrophic Factor; ERK: Extracellular signal-regulated kinase; mTOR: mammalian target of rapamycin; MAO: Monoamine oxidases; TrkB: Tropomyosin receptor kinase B; PI3K: Phosphatidylinositol-3 kinase; AKT: Serine/threonine-protein kinase; Nrf2: nuclear factor erythroid 2-related factor 2; NF-κB: Nuclear factor-κB; CNTF: Ciliary neurotrophic factor; CNTFRα: CNTF receptor alpha; JAK2: Janus kinase 2; STAT3: Signal transducers and activators of transcription 3; SIRT1: Silent information regulator 1; PGC-1a: Peroxisome proliferator-activated receptor gamma coactivator 1-alpha; APP: Amyloid precursor protein; PS1: Presenilin 1; TNF-α: Tumor necrosis factor-alpha; MMP-9: Matrix metalloproteinase-9.

**Table 2:** Pharmacological properties of *Apium graveolens* in neurological disorders (in vitro).

Cell	Intervention Details	Disease Model	Dosage, Duration	Effect/Mechanism	Reference
PC12	DL-NBP	Parkinson’s disease	0.01, 0.1, 1.0, 10 or 100 µM, 4 h	Accumulation of alpha-synuclein was diminished by L-NBP, which also decreased the formation of ROS and NO which indicate cytoprotection through inhibition of oxidative stress	Huang, 2010 [46]
PC12	NBP	Parkinson’s disease	0.1 M, 72 h	Groups that received NBP have a majority of their dendritic processes around maintained, indicating neuronal cells protection	Liu, 2012 [47]
Rat hippocampal neurons and SH-SY5Y human neuroblastoma	L-NBP	Parkinson’s disease	0.1, 1, 10 μM, 4 h	L-NBP guard neurons from harm brought on by Aβ -induced damage, possibly through preventing tau protein hyperphosphorylation.	Peng, 2008 [49]
PC12	DL-NBP	Diabetes (cognitive decline)	10 μM, 24 h	DL-NBP possibly acts on Nrf2 signaling pathway to alleviates oxidative stress and PI3K/Akt pathways, which are essential to enhance brain-derived neurotrophic factor expression levels	Wang, 2021 [36]
iPSC-VPC	DL-NBP	Stroke	10 μM, 48 h	DL-NBP significantly increased the expression of newly formed vascular marker PDGFR, SERCA2 and GLUT-1	Wei, 2021 [37]
Spinal motor neuron and SH-SY5Y human neuroblastoma	L-NBP	Charcot–Marie–Tooth disease	10 and 100 μmol/L, pre-treatment and treatment	Protective effects of L-NBP against mutation of HSPB8 caused by mitochondrial dysfunction	Yang, 2017 [48]

Abbreviations. ROS: Reactive oxygen species; NO: Nitric oxide; Nrf2: nuclear factor erythroid 2–related factor 2; PI3K: Phosphoinositide 3-kinase; Akt: Protein kinase B; PDGFR: Platelet-derived growth factor receptors; SERCA2: sarco/endoplasmic reticulum Ca^2+^-ATPase; GLUT-1: Glucose transporter 1, HSPB8: Heat shock protein B8.

## Data Availability

Not applicable.

## Supplementary Materials

The following supporting information can be downloaded at: https://www.mdpi.com/article/10.3390/molecules28155824/s1, Table S1: PRISMA checklist; Table S2: Search strategy for each electronic database; Table S3: Qualitative, quantitative and standardization details of *Apium graveolens* interventions. Reference [79] is cited in the supplementary materials.
