# Supplementary material for: Neurological Applications of Celery (Apium graveolens): A Scoping Review
Source: Molecules. 2023 Aug 2;28(15):5824. doi: 10.3390/molecules28155824 (PMC10420906; doi:10.3390/molecules28155824)
Supplement: Supplementary file 1 [file molecules-28-05824-s001.zip › Table S2 - Search strategy.pdf]

Supplementary Material

Table S2: Search strategy for each electronic database.

Database	Keyword search strategy	Year in search
PubMed	("apium"[MeSH Terms] OR "apium"[All Fields] OR ("apium"[All Fields] AND "graveolens"[All Fields]) OR "apium graveolens"[All Fields] OR (("apium"[MeSH Terms] OR "apium"[All Fields]) AND "celleri"[All Fields]) OR (("apium"[MeSH Terms] OR "apium"[All Fields]) AND "decumbens"[All Fields]) OR (("apium"[MeSH Terms] OR "apium"[All Fields]) AND ("dulce"[All Fields] OR "dulces"[All Fields])) OR ("apium"[MeSH Terms] OR "apium"[All Fields]) OR (("apium"[MeSH Terms] OR "apium"[All Fields]) AND "lobatum"[All Fields]) OR (("apium"[MeSH Terms] OR "apium"[All Fields]) AND "lusitanicum"[All Fields]) OR (("apium"[MeSH Terms] OR "apium"[All Fields]) AND "maritimum"[All Fields]) OR (("apium"[MeSH Terms] OR "apium"[All Fields]) AND "palustre"[All Fields]) OR (("apium"[MeSH Terms] OR "apium"[All Fields]) AND "rapaceum"[All Fields]) OR (("apium"[MeSH Terms] OR "apium"[All Fields]) AND "vulgare"[All Fields])) AND ("neurodegenerative diseases"[MeSH Terms] OR ("neurodegenerative"[All Fields] AND "diseases"[All Fields]) OR "neurodegenerative diseases"[All Fields] OR ("neurodegenerative"[All Fields] AND "disease"[All Fields]) OR "neurodegenerative disease"[All Fields] OR ("cognition disorders"[MeSH Terms] OR ("cognition"[All Fields] AND "disorders"[All Fields]) OR "cognition disorders"[All Fields]) OR ("central nervous system diseases"[MeSH Terms] OR ("central"[All Fields] AND "nervous"[All Fields] AND "system"[All Fields] AND "diseases"[All Fields]) OR "central	1960–2022

Database	Keyword search strategy	Year in search
	nervous system diseases"[All Fields] OR ("central"[All Fields] AND "nervous"[All Fields] AND "system"[All Fields] AND "disorders"[All Fields]) OR "central nervous system disorders"[All Fields]) OR ("nervous system diseases"[MeSH Terms] OR ("nervous"[All Fields] AND "system"[All Fields] AND "diseases"[All Fields]) OR "nervous system diseases"[All Fields] OR ("neurological"[All Fields] AND "disorder"[All Fields]) OR "neurological disorder"[All Fields]) OR ("brain injuries"[MeSH Terms] OR ("brain"[All Fields] AND "injuries"[All Fields]) OR "brain injuries"[All Fields] OR ("brain"[All Fields] AND "injury"[All Fields]) OR "brain injury"[All Fields]) OR ("stroke"[MeSH Terms] OR "stroke"[All Fields] OR "strokes"[All Fields] OR "stroke s"[All Fields]) OR ("alzheime s"[All Fields] OR "alzheimer disease"[MeSH Terms] OR ("alzheimer"[All Fields] AND "disease"[All Fields]) OR "alzheimer disease"[All Fields] OR "alzheimer"[All Fields] OR "alzheimers"[All Fields] OR "alzheimer s"[All Fields] OR "alzheimers s"[All Fields]) OR ("dementia"[MeSH Terms] OR "dementia"[All Fields] OR "dementias"[All Fields] OR "dementia s"[All Fields]) OR ("motor neuron disease"[MeSH Terms] OR ("motor"[All Fields] AND "neuron"[All Fields] AND "disease"[All Fields]) OR "motor neuron disease"[All Fields]) OR ("multiple sclerosis"[MeSH Terms] OR ("multiple"[All Fields] AND "sclerosis"[All Fields]) OR "multiple sclerosis"[All Fields]) OR ("amyotrophic lateral sclerosis"[MeSH Terms] OR ("amyotrophic"[All Fields] AND "lateral"[All Fields] AND "sclerosis"[All Fields]) OR "amyotrophic lateral sclerosis"[All Fields]) OR ("motor neuron disease"[MeSH Terms] OR ("motor"[All Fields] AND "neuron"[All Fields] AND "disease"[All Fields]) OR "motor neuron disease"[All Fields] OR ("primary"[All Fields] AND "lateral"[All Fields] AND "sclerosis"[All Fields]) OR "primary lateral sclerosis"[All Fields]) OR ("bulbar palsy,	

Database	Keyword search strategy	Year in search
	progressive"[MeSH Terms] OR ("bulbar"[All Fields] AND "palsy"[All Fields] AND "progressive"[All Fields]) OR "progressive bulbar palsy"[All Fields] OR ("progressive"[All Fields] AND "bulbar"[All Fields] AND "palsy"[All Fields])) OR ("pseudobulbar palsy"[MeSH Terms] OR ("pseudobulbar"[All Fields] AND "palsy"[All Fields]) OR "pseudobulbar palsy"[All Fields]) OR ("muscular atrophy, spinal"[MeSH Terms] OR ("muscular"[All Fields] AND "atrophy"[All Fields] AND "spinal"[All Fields]) OR "spinal muscular atrophy"[All Fields] OR ("progressive"[All Fields] AND "muscular"[All Fields] AND "atrophy"[All Fields]) OR "progressive muscular atrophy"[All Fields]) OR ("muscular atrophy, spinal"[MeSH Terms] OR ("muscular"[All Fields] AND "atrophy"[All Fields] AND "spinal"[All Fields]) OR "spinal muscular atrophy"[All Fields] OR ("spinal"[All Fields] AND "muscular"[All Fields] AND "atrophy"[All Fields])) OR ("bulbo spinal atrophy, x linked"[MeSH Terms] OR ("bulbo spinal"[All Fields] AND "atrophy"[All Fields] AND "x linked"[All Fields]) OR "x-linked bulbo-spinal atrophy"[All Fields] OR ("kennedy s"[All Fields] AND "disease"[All Fields]) OR "kennedy s disease"[All Fields]) OR ("spinal cord injuries"[MeSH Terms] OR ("spinal"[All Fields] AND "cord"[All Fields] AND "injuries"[All Fields]) OR "spinal cord injuries"[All Fields] OR ("spinal"[All Fields] AND "cord"[All Fields] AND "injury"[All Fields]) OR "spinal cord injury"[All Fields]) OR ("friedreich ataxia"[MeSH Terms] OR ("friedreich"[All Fields] AND "ataxia"[All Fields]) OR "friedreich ataxia"[All Fields]) OR ("huntington disease"[MeSH Terms] OR ("huntington"[All Fields] AND "disease"[All Fields]) OR "huntington disease"[All Fields] OR ("huntington s"[All Fields] AND "disease"[All Fields]) OR "huntington s disease"[All Fields]) OR ("lewy body disease"[MeSH Terms] OR ("lewy"[All Fields] AND "body"[All Fields] AND "disease"[All Fields]) OR "lewy	

Database	Keyword search strategy	Year in search
	body disease"[All Fields]) OR ("parkinson disease"[MeSH Terms] OR ("parkinson"[All Fields] AND "disease"[All Fields]) OR "parkinson disease"[All Fields] OR "parkinson s disease"[All Fields]) OR "paraplegi*"[All Fields] OR "quadriplegi*"[All Fields] OR ("neurologic manifestations"[MeSH Terms] OR ("neurologic"[All Fields] AND "manifestations"[All Fields]) OR "neurologic manifestations"[All Fields] OR ("neurological"[All Fields] AND "impairment"[All Fields]) OR "neurological impairment"[All Fields]) OR ("neurologic manifestations"[MeSH Terms] OR ("neurologic"[All Fields] AND "manifestations"[All Fields]) OR "neurologic manifestations"[All Fields] OR ("neurological"[All Fields] AND "deficit"[All Fields]) OR "neurological deficit"[All Fields]) OR ("cerebral palsy"[MeSH Terms] OR ("cerebral"[All Fields] AND "palsy"[All Fields]) OR "cerebral palsy"[All Fields]) OR ("brain ischaemia"[All Fields] OR "brain ischemia"[MeSH Terms] OR ("brain"[All Fields] AND "ischemia"[All Fields]) OR "brain ischemia"[All Fields]) OR "neuroprotect*"[All Fields] OR "cognit*"[All Fields] OR "neuroinflammat*"[All Fields] OR ("excitotoxic"[All Fields] OR "excitotoxically"[All Fields] OR "excitotoxicity"[All Fields] OR "excitotoxity"[All Fields]) OR ("excitotoxic"[All Fields] OR "excitotoxically"[All Fields] OR "excitotoxicity"[All Fields] OR "excitotoxity"[All Fields]) OR ("blood brain barrier"[MeSH Terms] OR ("blood brain"[All Fields] AND "barrier"[All Fields]) OR "blood brain barrier"[All Fields] OR ("blood"[All Fields] AND "brain"[All Fields] AND "barrier"[All Fields]) OR "blood brain barrier"[All Fields]) OR ("neurogenesis"[MeSH Terms] OR "neurogenesis"[All Fields] OR "neurogeneses"[All Fields]))	
Web of Science	(ALL=(Apium graveolens OR Apium celleri OR Apium decumbens OR Apium dulce OR Apium integrilobum OR Apium	1960–2022

Database	Keyword search strategy	Year in search
	lobatum OR Apium lusitanicum OR Apium maritimum OR Apium palustre OR Apium rapaceum OR Apium vulgare OR celery OR celeries OR celeriac OR celeriacs)) AND ALL=(medicinal OR medicinally OR medicinals OR medicine OR medicines OR therapeutic OR therapy OR therapeutical OR therapeutically OR therapeuticals OR therapeutics OR effect OR effecting OR effective OR effectively OR effectiveness OR effectivenesses OR effectives OR effectivities OR effectivity OR effects OR properties OR property OR bioactivate OR bioactivated OR bioactivates OR bioactivating OR bioactivation OR bioactivations OR bioactives OR bioactivities OR bioactivity OR efficacies OR efficacious OR efficaciously OR efficaciousness OR efficacy OR safety OR safeties OR harm OR benefit OR benefits)	
CENTRAL & LILAC	Any publications related to '*Apium graveolens*'	2000–2022
