# Supplementary material for: Neurological Applications of Celery (Apium graveolens): A Scoping Review
Source: Molecules. 2023 Aug 2;28(15):5824. doi: 10.3390/molecules28155824 (PMC10420906; doi:10.3390/molecules28155824)
Supplement: Supplementary file 1 [file molecules-28-05824-s001.zip › Table S3 - Qualitative, quantitative and standardization details of herbal interventions.pdf]

Supplementary Material

Table S3: Qualitative, quantitative and standardization details of *Apium graveolens* interventions.

No.	Author / Year	Disease / pharmacological action studied	Intervention formulation	Voucher specimen deposition	Qualitative analysis of content	Quantitative analysis of content	Standardization of intervention
1	Abu-Taweel, 2020	Perinatal effect	Details of celery not mentioned	No	No	No	No
2	Boonruamkaew, 2017	Depression	Crude 70% methanol extract of *A. graveolens*	Yes	Yes. HPLC	Yes. HPLC	No
3	Chen, 2021	Depression	DL-NBP	No	No	No	No
4	Chonpathompikunlert, 2018	Parkinson's disease	70% methanol crude extract of *A. graveolens* whole plant	Yes	Yes. HPLC	Yes. HPLC	Yes
5	Choupankareh, 2018	Epilepsy	Aqueous extract of *A. graveolens*	Yes, but no reference number	No	No	No

No.	Author / Year	Disease / pharmacological action studied	Intervention formulation	Voucher specimen deposition	Qualitative analysis of content	Quantitative analysis of content	Standardization of intervention
			aerial part				
6	Li, 2019	Ischemic stroke	DL-NBP	No	No	No	No
7	Li, 2020	Chronic cerebral hypoperfusion (CCH)	DL-NBP	No	No	No	No
8	Min, 2014	Chronic intermittent hypoxia hypercapnia	DL-NBP	No	No	No	No
9	Peng, 2010	Alzheimer's disease	L-NBP	No	No	No	No
10	Peng, 2012	Alzheimer's disease	L-NBP	No	No	No	No
11	Roghani, 2009	Diabetes (learning memory)	Aqueous extract of *A. graveolens*	No	No	No	No
12	Wang, 2021	Diabetes (cognitive decline)	DL-NBP	No	No	No	No

No.	Author / Year	Disease / pharmacological action studied	Intervention formulation	Voucher specimen deposition	Qualitative analysis of content	Quantitative analysis of content	Standardization of intervention
13	Wei, 2021	Stroke	DL-NBP	No	No	No	No
14	Wongtawatchai, 2017	Anxiety	Methanol exact of *A. graveolens* whole plant	Yes	Yes, method unclear	Yes. Folin-Ciocalteu colorimetric method	No
15	Xiang, 2014	Alzheimer's disease	L-NBP	No	No	No	No
16	Yang, 2018	Depression	DL-NBP	No	No	No	No
17	Ye, 2018	Epilepsy	L-NBP	No	No	No	No
18	Yuan, 2022	Neurotoxicity	NBP	No	No	No	No
19	Zeng, 2020	Stroke	L-NBP	No	No	No	No
20	Zhang, 2012	Stroke	DL-NBP	No	No	No	No
21	Zhang, 2016	Alzheimer's disease	L-NBP	No	No	No	No

No.	Author / Year	Disease / pharmacological action studied	Intervention formulation	Voucher specimen deposition	Qualitative analysis of content	Quantitative analysis of content	Standardization of intervention
22	Huang, 2010	Parkinson's disease	DL-NBP	No	No	No	No
23	Liu, 2012	Parkinson's disease	NBP	No	No	No	No
24	Peng, 2008	Parkinson's disease	L-NBP	No	No	No	No
25	Yang, 2017	Charcot–Marie–Tooth disease	L-NBP	No	No	No	No
26	Zhou, 2019	Parkinson's disease	NBP	No	No	No	No

Abbreviations. DL & L: ways of denoting enantiomers; NBP: 3-n-butylphthalide; HPLC: high performance liquid chromatography

Note. *Unclear: mentioned methods done similar as a previous study but not mentioned whether the qualitative analysis was done in the current study.
